# Electrochemotherapy in the Treatment of Bone Metastases: A Phase II Trial

**DOI:** 10.1007/s00268-016-3627-6

**Published:** 2016-07-21

**Authors:** Giuseppe Bianchi, Laura Campanacci, Mattia Ronchetti, Davide Donati

**Affiliations:** 1 Clinica Ortoepdica III, Istituto Ortopedico Rizzoli, Bologna, Italy; 2 Oncology Clinical, Research & Development, IGEA S.p.A., via Parmenide 10/a, 41012 Carpi, Modena Italy

## Abstract

**Introduction:**

Bone metastatic disease is a major cause of pain and decreased quality of life in patients with cancer. In addition to systemic therapy and pain control with narcotic analgesics, standard local treatments include palliation with radiation therapy and surgery. However, 20–30 % of patients do not respond to conventional treatments, increasing the interest in alternative therapies. We present the results of a new minimally invasive technique in the treatment of bone metastases.

**Methods:**

Twenty-nine patients affected by painful bone metastases were treated with electrochemotherapy (ECT) from July 2009 to July 2011; the mean age was 60 years (range 37–87); 21 patients received a previous ineffective local treatment; the appendicular skeleton was affected in 15 patients while in 14 patients other sites were involved. ECT was performed using the Cliniporator Vitae under fluoroscopy or CT guidance depending on the site of the lesion. Clinical response was assessed using VAS scale and objective tumour response was evaluated according to the MD Anderson criteria for bone metastases.

**Results:**

All patients well tolerated the procedure and no intraoperative or postoperative complications were observed. At a mean follow-up of 7 months, 24 patients were available for evaluation. 84 % of the patients (20 out of 24) referred improvement of pain ≥50 % with reduction of narcotics consumption. Radiographic evaluation after 3 months in 20 evaluable patients, showed “partial response” in 1 patient, “stable disease” in 17 and “progression” in two cases.

**Discussion:**

Results reported in this study demonstrated ECT to be safe and feasible in the treatment of painful bone metastases even when other previous treatments were ineffective. Pain and disease progression control was achieved in the majority of the patients with consequent improvement of quality of life.

**Conclusion:**

ECT should be considered a new feasible tool in the treatment of bone metastases in place or in combination with standard treatments; further developments are required to extend the use of this technique to spine metastases.

## Introduction

Prolonged cancer survival achieved with advances in medical therapies has increased significantly the number of patients with painful bone metastases, requiring new treating modalities in order to improve quality of life [1–3].

Surgery treatment is often required to prevent or treat pathologic fractures of long bones or to release spinal cord compression in case of spine metastases [4] with the aim of function preservation and quick mobilisation of the patient. When surgery is not indicated or in completion to surgery, radiation therapy in fractionated doses (8 Gy/1f or 30 Gy/10f) is considered the standard treatment but could be ineffective in 30–50 % of the cases.

Other minimally invasive technique are available (radiofrequency thermal ablation or selective arterial embolization) but with similar or worst in term of local progression or pain control. [1].

Electrochemotherapy (ECT) refers to the combination of chemotherapy and local delivery of electric pulses to the tumour nodule. Electric pulses induce cell membrane electroporation to increase drug diffusion into the cell and its cytotoxicity; bleomycin is the drug used for ECT as its activity in vivo is increased >80 times following membrane electroporation. ECT has proven effective in the treatment of metastases from solid tumours of any histology located to the skin or subcutaneous tissue. The multicenter study ESOPE (European Standard Operating Procedure for ECT) demonstrated that ECT may achieve an objective response rate of 85 % (74 % complete response rate) in treated tumour lesions [5, 6]. Furthermore, in nodules undergoing complete response, long-term histology shows that no residual tumour cells are detected locally [7–9].

Following extensive clinical studies confirming safety and efficacy of ECT, clinicians and researchers developed new strategies to extend its use to the treatment of non-superficial tumours [10].

Encouraging results reported in preclinical studies and in animal models demonstrated electroporation does not alter bone mineral structure, regenerative activity and mechanical competence [11–13] providing preclinical basis for use of ECT in the treatment of metastasis to bone in patients.

As a first in human application of ECT in the treatment of bone metastases, this prospective study was designed and conducted with the primary endpoints of feasibility and safety.

## Patients and methods

A prospective, single centre, single arm Phase II clinical trial was approved by the ethical committee of our institution (Prot.n.12458/15.05.2009—ES2IOR). All patients gave their written, informed consent to participate in the study.

Eleven patients enrolled in the study were grouped in Group 1 from July 2009 to July 2011. After completing the treatment of patients of Group 1, besides that, a second Group of 18 patients (Group 2) with tumour larger than 6 cm was evaluated (protocol amendment).

Study primary endpoints were feasibility and safety of ECT when applied to metastases to the bone; secondary endpoints were patients' clinical outcome and changes of metastases at imaging investigations.

Feasibility was assessed by evaluating the possibility of reliably inserting electrodes percutaneously in bone, according to a predefined geometry to ensure proper electroporation of cell membranes in the bone metastasis, hence to successfully complete the procedure.

For safety, all adverse events, after treatment and at follow-up, were recorded and evaluated according to CTCAE criteria (Common Toxicity Criteria for Adverse Events). We considered severe events those that would require substantial medical intervention in consequence of the local treatment with ECT.

Inclusion criteria were histologically proven involvement of appendicular skeleton by metastatic carcinoma or melanoma; maximum metastasis length 6 cm; no local treatment in previous 3 months; life expectancy >3 months; Karnofsky 70 %.

To assess tumour volume/density, all patients underwent standard X-ray, MRI or CT scan, with contrast enhancement, at presentation. Patients with multiple lesions were considered for enrolment if a target symptomatic lesion could be identified. Each case was discussed at institutional multidisciplinary meeting.

Exclusion criteria were patients with pathological fractures, with symptomatic or rapidly progressive visceral disease, known allergic reaction to bleomycin, cumulative bleomycin dose exceeding 250 µ/m^2^, abnormal haemostasis, chronic renal dysfunction, arrhythmia, pregnancy or lactation.

Protocol foresaw follow-up visits at 2 months for clinical evaluation and at 3 months for CT scan. Further clinical evaluations were scheduled at 6 months and 1 year. Clinical response included evaluation of change in pain by the VAS scale; use of pain killers, quality of nights sleep and return to daily activities [14].

Objective tumour response was evaluated according to the MD Anderson (MDA) criteria that define four different categories of response (CR-complete response, PR-partial response, PD-progressive disease and SD-stable disease) [15]. Radiological and clinical evaluations were performed by two investigators, GB and LC.

Demographic, tumour specifications and site of involvement regarding the two groups are reported in Table 1.

**Table 1:** Demographic, tumour specifications and site of involvement

	Group 1	Group 2
Patients	11	18
Gender (M/F)	4/7	6/12
Mean age (range)	60 (38–69)	61 (37–87)
Primary tumour		
Kidney	3	6
Melanoma	2	0
Prostate	1	0
Breast	1	4
Thyroid	2	1
Lung	1	0
Colon	1	3
Metastatic sarcoma	0	1
Bladder	0	1
Endometrioid carcinoma	0	1
Site of involvement		
Femur	6	2
Pelvis	2	7
Scapula	1	0
Tibia	1	4
Sacrum	0	3
Humerus	1	1
Previous local treatment		
Radiotherapy	6	11
Embolization	1	2
HiFu	0	1

**Fig. 1:**
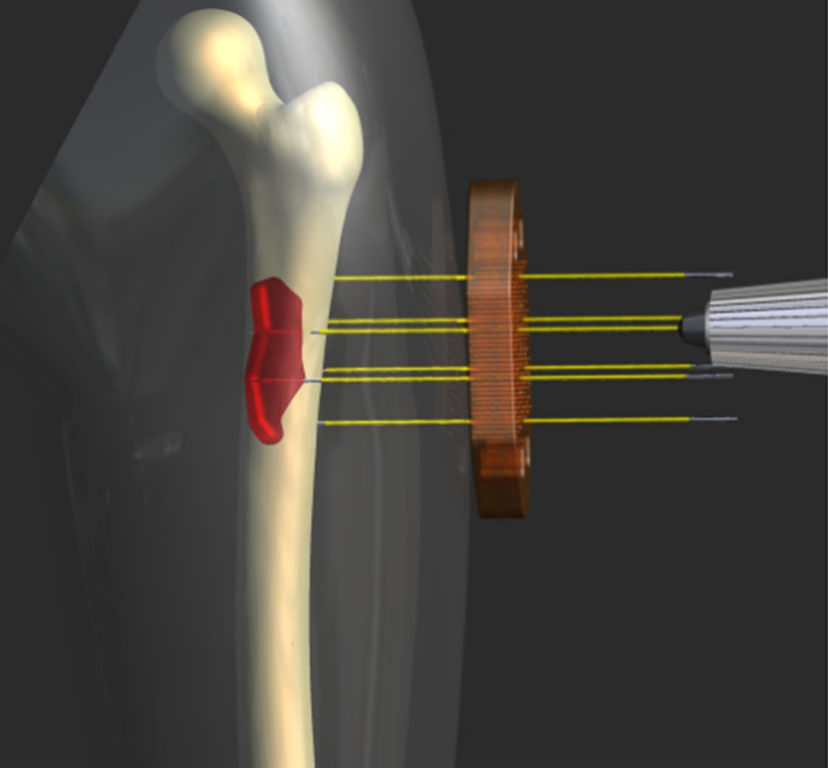
Schematic representation of electrode insertion in proximal femur: electrodes and drill; positioning mask on skin surface; bone metastases in *red*

Tumour cell electroporation was performed using the Cliniporator VITAE (IGEA S.p.A., Carpi, Italy). I 20 cm insulated needle electrodes (3 or 4 cm active tip) were drilled into the healthy bone to surround the metastases (Fig. 1). The number of needles used for each session varied from 6 to 8 according to tumour size and geometry. For electrode insertion, fluoroscopy guidance was used in 16 patients with peripheral skeleton involvement (7 femur, 5 tibia, 3 humerus, 1 scapula), while in other 14 patients with pelvic (9), sacral (4), femoral (1) lesions CT guiding was preferred. The choice of imaging technique used for electrode insertion was left at the discretion of the investigators, based on lesion accessibility.

ECT procedures were performed according to ESOPE study; briefly: 8 pulses of 1000 V/cm were delivered between each couple of electrodes to homogeneously cover the metastases with local electric field (>350 V/cm) to induce cell membrane electroporation. Bleomycin, 15 µ/m^2^ (Bleomycin Nippon Kayaku, Sanofi Aventis, Milan, Italy), was administrated intravenously in 8 min bolus before applying the electric pulses. This time is required to allow the distribution of the drug in all interstitial spaces at an effective concentration. The treatment has to be completed within 28 min from bleomycin injection [16]. As per protocol, patients could undergo up to 2 ECT treatments, 4 weeks apart, based on the possibility of increasing local disease control [6]. Further treatments were offered to patients that had significant benefit from ECT and had pain recurrence at the same site.

In 14 patients (48 %), the treatment was completed under regional anaesthesia and in 16 patients (52 %) under general anaesthesia. Patients' general conditions influence the choice of anaesthesia protocol, when possible regional anaesthesia was preferred. Type of anaesthesia does not influence the ECT treatment outcome [6].

## Results

**Fig. 2:**
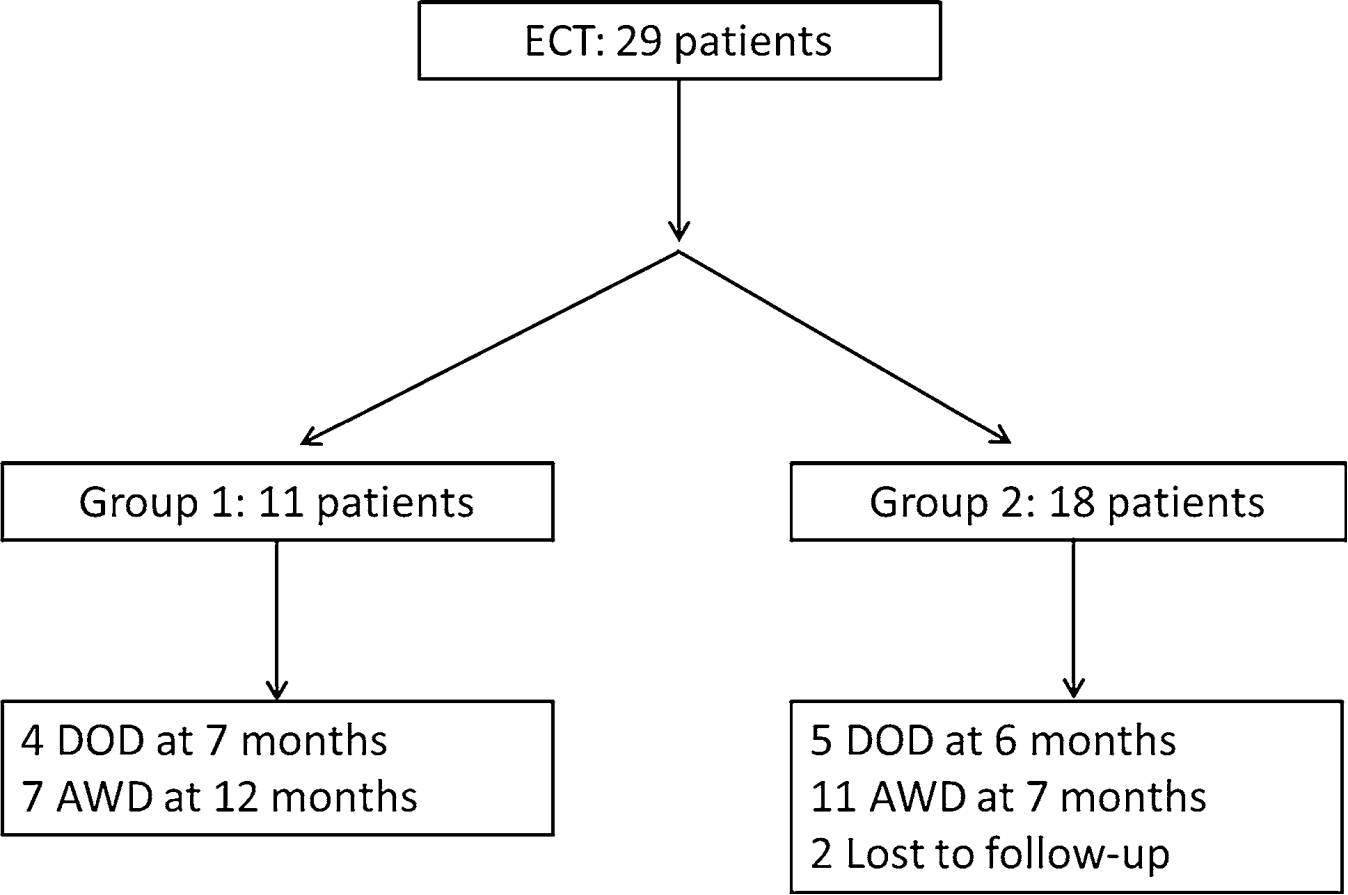
Organogram of patients treated. Group 1 patients per protocol; Group 2 compassionate treatment. *DOD* dead of disease, *AWD* alive with disease

Overall 29 patients successfully underwent at least one ECT procedure and they were discharged from the hospital the day after ECT treatment without complications. 43 procedures were completed in 29 patients. Mean follow-up was 7 months (1–22), two patients were lost at follow-up, nine died because of the disease and 18 were alive with disease at last follow-up (Fig. 2). No acute side effects related to bleomycin injection were observed.

### Primary outcome

In group 1, the procedure was repeated four times in one patient, three times in another patient, two times in two patients and the other seven patients received only one procedure. Patients receiving more than two ECT treatments did so after completing and exiting the clinical trial according to study protocol. Further treatments were offered to maintain local disease control when other options were exhausted.

In group 1, femur fracture occurred after the second treatment showing that repeated electrode insertion weakens the cortical bone. Nevertheless, the metastasis had not progressed and the fracture treated by endomedullary nailing healed. Histology of cells recovered at fracture site was negative for the presence of tumour cells.

Among patients in group 2, 12 patients received a single ECT treatment, five patients received two treatments and one patient received three treatments.

In both groups, no life threatening events were associated with the treatment. Nevertheless, two severe adverse events that occurred in Group 2 were investigated. A wide area of necrosis and ulceration, in previously irradiated skin of proximal tibia, required above knee amputation. Neurogenic bladder was observed after the third treatment of a large lesion involving the sacrum. Neurogenic bladder did not recover spontaneously, and it was deemed related to local disease progression.

### Secondary outcome

In group 1, clinical results were available for eight patients only. The mean pre-ECT VAS value was 5 (2–10) that, after ECT lowered to 2 (0–4). Significant pain decrease (≥50 %) was reported by six out of eight patients.

Eight patients diminished the use of pain killers and improved the quality of night’s sleep; daily activities ameliorated in six patients in long-term follow-up.

For seven patients, additional diagnostic imaging was available at more than 3 months follow-up showing partial response in one patient and stable disease in six patients. Four patients were dead.

In group 2, clinical results were available for 16 patients as two patients did not return for follow-up visit. The mean pre-ECT VAS value was 7 (2–10) and declined to 3 (0–8) after ECT. Pain decrease ≥50 % was reported in 14 out of 16 patients.

Diagnostic imaging at more than 3 months follow-up was available for 13 patients: 11 showing stable disease and two patients showing progressive disease.

**Fig. 3:**
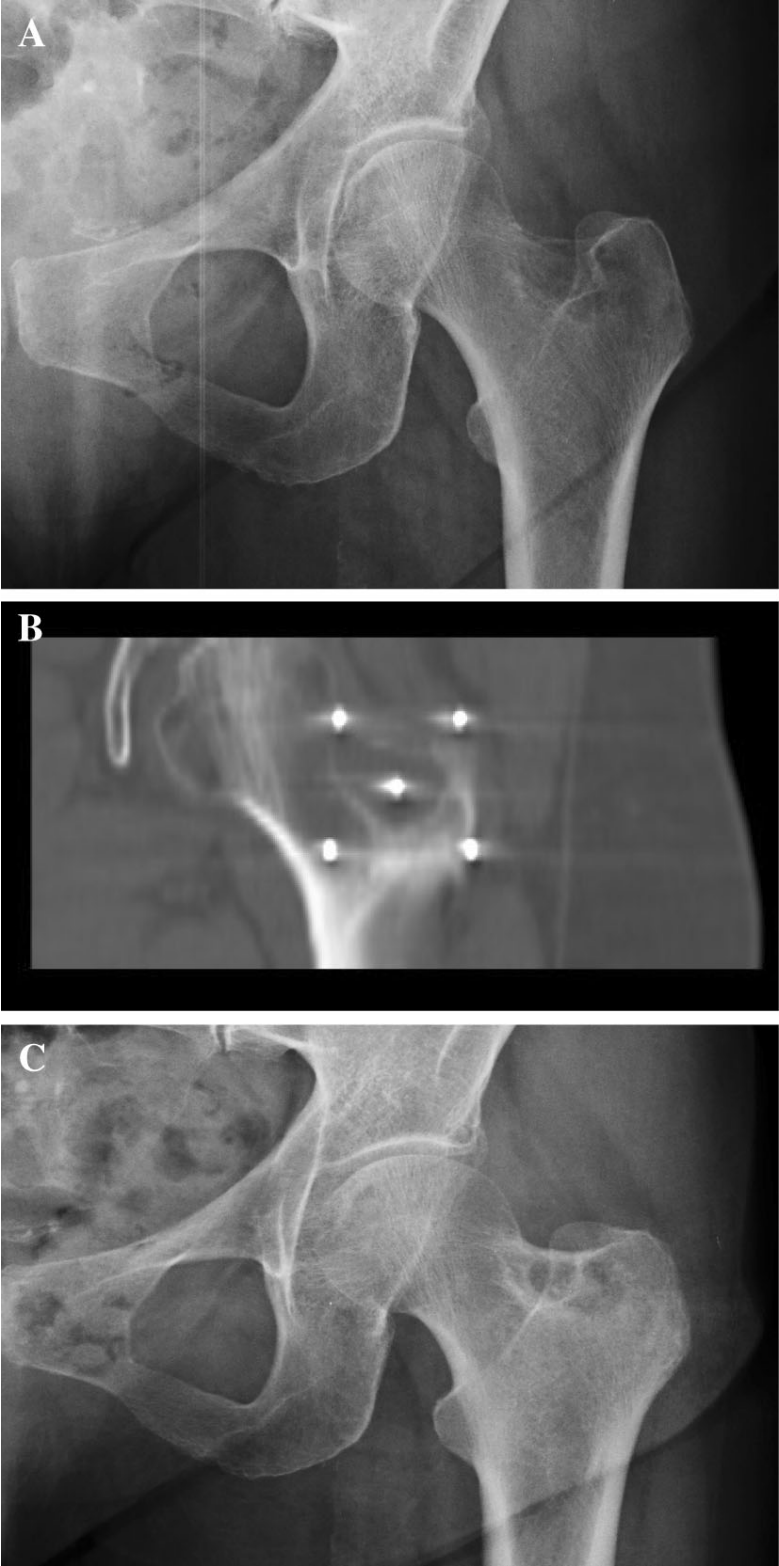
**a**–**c** A 69-year-old patient: left femoral neck painful bone metastases, thyroid carcinoma, **a** CT-guided ECT needles positioning, coronal plane, **b** X-ray result at 6 months of follow-up; sclerotic rim surrounding the lesion: Partial Response (**c**)

**Fig. 4:**
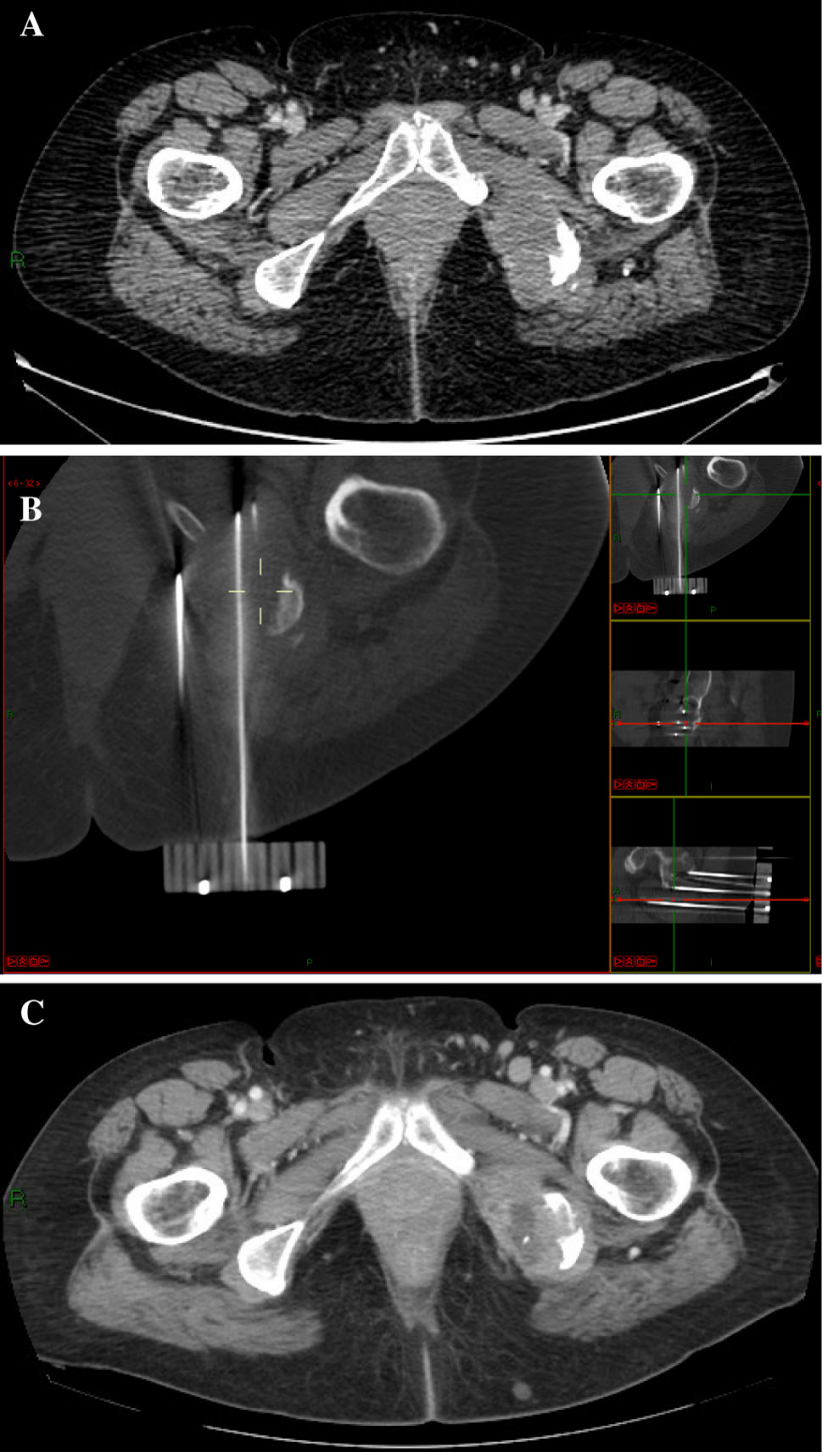
**a**–**c** A 57-year-old patient: left ischium painful bone metastases, kidney carcinoma, **a** CT-guided ECT needles positioning and multiplanar reconstruction, **b** CT scan at 7 months follow-up; central necrosis and shrinking of the metastatic lesion: Partial Response (**c**)

Figure 3 and 4 briefly sum up two CT-guided procedures and their radiographical results at follow-up.

## Discussion

To the best of the authors’ knowledge, this is the first prospective clinical study on the use of ECT for the treatment of bone metastasis even if results are reported on a small number of patients and with the lack of comparative arm.

Extensive evidence on the efficacy of ECT in metastasis to the skin and subcutaneous tissue of different histological origins is present in the literature [5, 10].

Preclinical data on the use of ECT for the treatment of metastases to bone provided the rationale for designing and conducting this clinical trial [11, 13]. We developed the procedure to treat ECT patients with bone metastases. The orthopaedic surgeon can perform the procedure without difficulties, no complications were experienced during the operating procedure. ECT can be performed in the proximity of vital structures considered there is no thermal denaturing effect; furthermore, applied electric pulses have no damaging effect on nerves and vessels [17, 18]. Treatment was easier to perform in patients with small and regularly shaped lesions.

Pain relief was achieved in 84 % of patients. Use of pain killers, quality of night’s sleep and daily activities improved in 55–73 % of patients. These results are relevant in this group of patient that already underwent treatments that proved to be ineffective (radiotherapy and/or SAE). These patients presented with deteriorate overall health status or severe co-morbidities and major surgery was not indicated. Local tumour control (stable disease) was achieved in most patients; only 10 % of the lesions showed progression at follow-up.

In patients treated according to protocol one, fracture that occurred in femur after a second treatment was explained by the repeated electrode insertion, that led to the weakening of bone mechanical competence.

In one patient of group 2, severe skin necrosis required leg amputation. Necrosis and ulceration should always be considered carefully in patients with poor soft tissue envelop or in area previously irradiated. We did not observe systemic toxic effects of bleomycin. Lung toxicity of bleomycin is well known, this occurs only when the cumulative dose exceeds 250–300 U much higher than the dose here used. Previous use of high doses of bleomycin was an exclusion criteria.

Almost 75 % of metastasis to bone are symptomatic and require local treatment to improve quality of life and prevent local disease progression. Except primary surgery, external beam radiotherapy is the main treatment modality for pain control with a response rate of 70 % [14, 19]. However, radiation therapy weakens bone mechanical competence and it affects bone blood supply, which can lead to post-radiation fracture requiring further treatment such as resection and reconstruction with a prosthesis [2].

Other treating modalities have been proposed and different results are reported according to treatment type. Goetz et Al. and Gronemeyer reported effective pain control with radiofrequency ablation (RFA) (75–95 %). Rossi et al. reported good results in pain-control using selective arterial embolization (SAE) in patients affected by highly vascularised metastatic lesions such as renal cancer metastases. In a series of 107 patients, pain control was achieved in 96 % of the cases but transient effect was frequent, requiring multiple treatments [20].

ECT is a new minimally invasive technique easily feasible at the appendicular skeleton and, with more difficulties, to other skeletal sites. Differently from other techniques, ECT should not be considered an “ablative” procedure, but a “neoplasm-selective” procedure, sparing healthy tissue from the toxic effect of bleomycin and preserving the mechanical competence of bone [13]. These characteristics suggest that ECT might be electively used for early control of isolated metastases to bone, to finally preserve bone quality, to favour bone reconstruction and avoid the surgical procedures required to protect bone from fracture. The results obtained in Group 2 indicate that ECT can play an important role in patients with advanced disease and inoperable metastases resulting in immediate pain relief and improvement in quality of life.

This study shows that the ECT procedure is safe and can spare patients major surgery. Compared to other ablation treatments, ECT is minimally invasive, repeatable and preserves mechanical competence of bone tissue; furthermore, other treatments are possible. Present limit of the technique is spine metastases treatment, since the vicinity of the spinal cord and technical limitations of electrode positioning.

## Conclusion

Results reported in this study demonstrated ECT to be a feasible, safe and effective technique in the treatment of painful bone metastases of the pelvis and appendicular skeleton, even when other previous treatments were ineffective. Pain and disease progression control was achieved in the majority of the patients with consequent improvement of quality of life. Further developments are required to extend the use of this technique to spine metastases.
